# Predicting collapse of adaptive networked systems without knowing the network

**DOI:** 10.1038/s41598-020-57751-y

**Published:** 2020-01-27

**Authors:** Leonhard Horstmeyer, Tuan Minh Pham, Jan Korbel, Stefan Thurner

**Affiliations:** 1 Section for the Science of Complex Systems, CeMSIIS, Medical University of Vienna, Spitalgasse 23, A-1090 Vienna, Austria; 2 Complexity Science Hub Vienna, Josefstädterstrasse 39, A-1080 Vienna, Austria; 3 Santa Fe Institute, 1399 Hyde Park Road, Santa Fe, NM 87501 USA; 4 IIASA, Schlossplatz 1, 2361 Laxenburg, Austria

**Keywords:** Population dynamics, Applied mathematics, Physics

## Abstract

The collapse of ecosystems, the extinction of species, and the breakdown of economic and financial networks usually hinges on topological properties of the underlying networks, such as the existence of self-sustaining (or autocatalytic) feedback cycles. Such collapses can be understood as a massive change of network topology, usually accompanied by the extinction of a macroscopic fraction of nodes and links. It is often related to the breakdown of the last relevant directed catalytic cycle within a dynamical system. Without detailed structural information it seems impossible to state, whether a network is robust or if it is likely to collapse in the near future. Here we show that it is nevertheless possible to predict collapse for a large class of systems that are governed by a linear (or linearized) dynamics. To compute the corresponding early warning signal, we require only non-structural information about the nodes’ states such as species abundances in ecosystems, or company revenues in economic networks. It is shown that the existence of a single directed cycle in the network can be detected by a “quantization effect” of node states, that exists as a direct consequence of a corollary of the Perron–Frobenius theorem. The proposed early warning signal for the collapse of networked systems captures their structural instability without relying on structural information. We illustrate the validity of the approach in a transparent model of co-evolutionary ecosystems and show this quantization in systems of species evolution, epidemiology, and population dynamics.

## Introduction

Complex networked systems are prone to collapse. This is the case for species evolution^1–3^ where collapse may lead to mass extinctions in ecosystems^4,5^, but it equally holds for economic systems^6,7^, the financial sector^8^, social networks^9^, or societies as a whole^10^. The prediction of collapse in large networked systems is extremely challenging, since stability and resilience may depend on specific details of the system that are hard to monitor. Collapse might arise as a consequence of the extinction of a single species in an ecosystem^11–14^, or the default of a single financial player^15–18^. Often systems are composed of many thousands of constituents and even more interactions. Whether the elimination of any node or interaction in a network leads to collapse depends to a large extent on the structural details of the interaction network.

Let us consider a minimal version of a networked dynamical system composed of *N* components, given by

1 $$\frac{d}{dt}{X}_{i}=\mathop{\sum }\limits_{j=1}^{N}\,{M}_{ij}{X}_{j}-\Phi {X}_{i},$$

where *X*_*i*_ is the state of the *i*-th component. Depending on the context, *X*_*i*_ could be populations of species, revenues of companies, abundances of molecules, or the degree of contagiousness of individuals. The growth of each *X*_*i*_ depends on the other states via the interaction matrix, *M*, whose entries *M*_*ij*_ determine to what extent *j* influences *i*. Self-interactions are excluded, *M*_*ii*_ = 0; Φ*X*_*i*_ is a depletion or decay term. We consider the interactions described by a Metzler matrix, i.e., *M*_*ij*_ ≥ 0. When representing *M* as an interaction network, a directed link is drawn from *j* to *i* if and only if *M*_*ij*_ ≠ 0. The influence of individual nodes can spread through the entire network via (1), which results in interdependencies that depend on the global network structure. More complex behaviour arises when the network *M* is co-evolving or when constraints for the dynamics are in place. Many systems are well described by adaptive linear models as in (1), however, the matrix *M* also evolves. Examples include catalytic networks, chemical reaction networks, aging processes, or economical models^19–21^. Many systems often behave effectively linear, especially when they evolve around an equilibrium or a quasi-stationary state. In ecological modelling, one often considers the slowly evolving degrees of freedom as effectively fixed and derives linear models for the fast variables^22^. Also linear epidemiological models are used when modeling situations close to an endemic state or just before the outbreak^23^.

It is a well known fact that the existence of cycles in the interaction network, i.e. directed closed paths whose only repeated vertices are the first and the last, is a crucial stability factor for systems of type (1) since they constitute self-reinforcing and self-sustaining structures. Conversely, the absence of cycles indicates a lack of self-sustaining mechanisms. For the minimal linear model (1) it is known that all states decay to zero for Φ > 0, when cycles are absent. A foundational underpinning of this argument was put forward by Manfred Eigen, who proposed autocatalytic cycles as a guiding principle for self-replication in nature, and a possible scenario for the origin of life^24^. These ideas were further developed in^25^ and later investigated in^26–28^, and were tested experimentally^29^. Other famous examples of self-sustaining cycles include the autocatalytic Bethe-Weizsäcker cycle of hydrogen burning in the sun^30^, or the Calvin cycle^31^ of photosynthesis in plants. The interaction structure of complex systems often evolves over time (co-evolution), and cycles may get created and destroyed in this process. This structural evolution is generally the result of an adaptation process^32^, and happens at a much slower rate than the dynamics of the nodes. For example, evolutionary processes are much slower than the population dynamics of the species, and new ties between companies are formed at a slower rate than the revenue generation from these ties. The dynamics in (1) constitutes a fast process on a static or quasi-static interaction network, given by the interaction matrix *M*. In this view, collapse is tightly related to the rupture of all cycles. A system with a single cycle is the most unstablebecause the deletion of any cycle-node or link breaks the sustaining feedback mechanism. The systemic relevance of a node can therefore be seen as the number of cycles that pass through it. Generally, the more cycles there are in a networked system, the more stable it becomes. If we understand the collapse of a network as a massive change of topology, accompanied by the extinction of a macroscopic fraction of nodes, we can define a *collapse* of a networked dynamical system as the transition from a system with cycles to one without cycles. This definition follows the definition of collapse in the Jain-Krishna model^33^, which is a prominent example of an adaptive linear networked model.

The central idea of this paper is to develop an early warning signal that detects the last surviving cycle in a networked dynamical system. We will show that—surprisingly—this is possible from the observation of the node states for systems of type (1) with binary (0, 1) entries in *M*. This result can be phrased as a mathematical theorem that extends a special case of the Perron–Frobenius theorem^34^. Its significance in this context has hitherto not been recognized. Intuitively, the detection of cycles requires the full knowledge of the interaction network, since a cycle could pass through only two nodes or through the entire network. The fact that the proposed warning signal only requires information about the node states has two advantages. First, for a networked system with *N* nodes, the information about the entire interaction network could require up to ${\mathscr{O}}({N}^{2})$ observations, which usually becomes unfeasible for large systems. Second, detailed network information is often not available or accessible. For the case it is available, there are many algorithms^35–38^ to find the cycles.

This type of structural precursor signal, that only relies on non-structural observations, is different from previously used precursors. Precursor signal theory has mostly focused on changes in time series close to a critical transition or a collapse point. In particular, classical precursor signals exploit the increasing auto-correlations^39^, increasing volatility, increasing relaxation times from perturbations^40^, and their scaling behaviour close to the transition^41^. These methods typically probe the stability of the fast changing processes and can not capture the presence of cycles. However, for systems with complex network structure the stability of the network itself is crucial, i.e. the stability with respect to a structural perturbation such as the deletion of nodes or links. Precursor signals that take into account the network structure directly are typically based on motif analysis^42^. Network motifs are local structures that do not capture the influence of the entire network. The risk of collapse can be severely misjudged from this partial knowledge, as has been recently demonstrated in the context of adaptive epidemiology^43^.

## Results

Consider a system that evolves as (1). We state our main result in the simplest formulation of a directed unweighted network *M*, which means that *M*_*ij*_ = 1, if the change of *i* depends on the change of *j*. In such systems, the normalized vector ${x}_{i}={X}_{i}/\sum _{j}\,{X}_{j}$ “quantizes” if there is one single cycle remaining in *M*. This means that all entries in *x*_*i*_ are multiples of some minimum value *x*_min_. This is captured in the following theorem:

**Theorem 1** (Eigenvector Quantization). *Let M be a binary matrix with entries M*_*ij*_ ∈ {0, 1} *and diagonal entries M*_*ii*_ = 0 *for all i* ∈ {1, …, *N*}. *Let G be the directed network with directed adjacency matrix M*. *Let X*(*t*) = (*X*_1_(*t*), …, *X*_*N*_(*t*)) *be an N-dimensional state vector*, *whose components X*_*i*_(*t*) *evolve according to* (1). *For all initial conditions X*(0), *except for a set of Lebesgue-measure zero*, *the normalised vector x*(*t*), *defined component-wise as* ${x}_{i}(t)={X}_{i}(t)/\sum _{j}\,{X}_{j}(t)$, *converges to a stable fixed point x:*= *lim*_*t*→∞_*x*(*t*), *for which the following holds*:

***Eigenvector Quantization:**** Suppose G contains only one single cycle*. *Then any component x*_*i*_ *can be expressed as*

2 $${x}_{i}={n}_{i}{x}_{{\rm{\min }}},$$

where *x*_min_ is the minimal non-zero component and *n*_*i*_ is a natural number. The value of *x*_*min*_ is taken by the cycle-nodes, and the integer *n*_*i*_ ≥ 0 is the number of directed paths that lead from cycle-nodes to node *i*. If there are no paths from cycle nodes, then *x*_*i*_ = 0.

**Figure 1:**
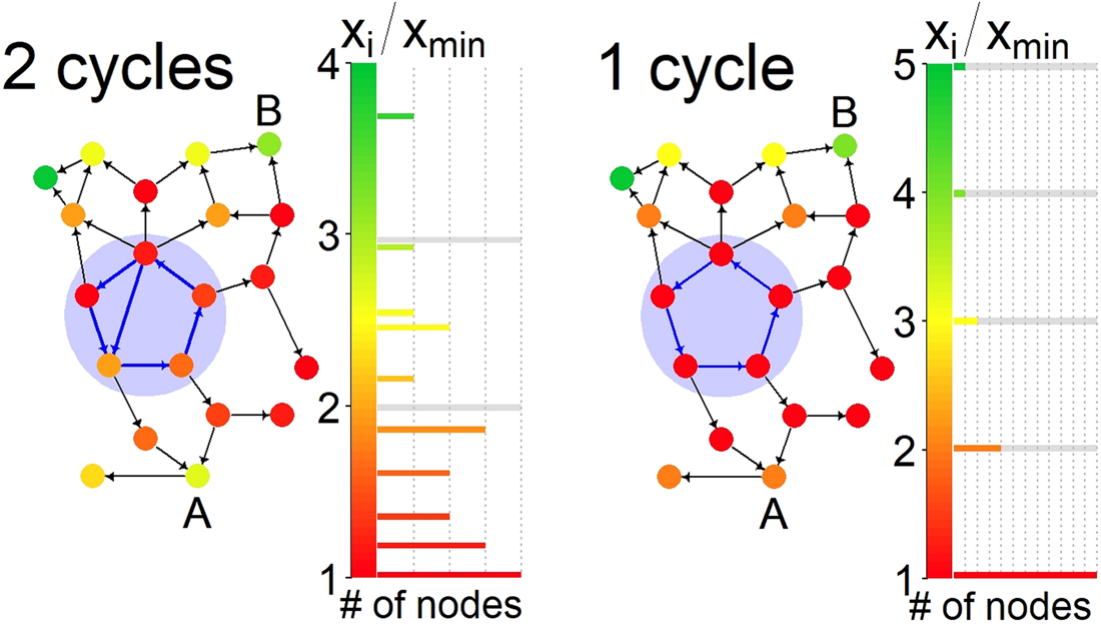
Graphical demonstration of the quantization theorem. Directed networks *M* containing two cycles (left) and one cycle (right). Cycles are in the shaded area. The node color indicates the state, *x*_*i*_, (value of the component of the state vector, *x*) in units of the minimal value *x*_min_. The histograms show the number of nodes in a given state. In the right-hand figure we see the quantization of states due to the presence of a single (!) remaining cycle. The occurrence of quantization at the last remaining cycle can be used as a precursor signal. The state *x*_*i*_/*x*_min_ in the single-cycle network (right) coincides with the number of directed paths from the cycle to node *i*. Node *A* can be reached through two paths from the cycle, while node *B* can be reached by four. For the multi-cycle case (left), the number of paths no-longer coincides with the states.

For the proof of the theorem, see (*SI*). Note that the theorem gives a sufficient condition for the eigenvector quantization of the node states. A graphical illustration of the theorem is shown in Fig. 1. We compare two networks that are identical except for one link. The network in Fig. 1 on the left-hand side contains two cycles, indicated by blue arrows, the network in Fig. 1 right-hand side has only one last remaining cycle, again indicated by blue arrows. The node color corresponds to the respective components of the normalized state vector, *x*, rescaled by the minimum value, *x*_min_. The histogram to the right of each network figure indicates the number of nodes, whose components share a given value of *x*/*x*_min_. It can be seen from the histogram in Fig. 1 right that the state vector takes only discrete, equidistant values when the network has one remaining cycle. Once the quantization occurs, one can immediately determine the number of paths by which a node can be reached from the cycle, *x*_*i*_/*x*_min_. Node *A* can be reached via two paths from the cycle, while node *B* can be reached via four. For the multi-cycle case (a), the number of paths no-longer coincides with the states.

The key mechanism behind quantization can be explained as follows. The state of a given node does not only depend on the nodes which feed directly into it, but through them, indirectly also depends on a large number of other nodes, and possibly the entire network. This global interdependence is best understood through the equation that is satisfied by the stable fixed point of the normalized state-vector *x* = lim_*t*→∞_*x*(*t*), namely the eigenvalue equation (Lemma 1 in *SI*):

3 $${\lambda }_{1}{x}_{i}=\sum _{j}\,{M}_{ij}{x}_{j},$$

where *λ*_1_ is the maximal eigenvalue of *M*. This equation couples all node states. It is a well-known fact that *λ*_1_ = 1 if the network contains only one cycle^34^. In this case ${x}_{i}=\sum _{j}\,{M}_{ij}{x}_{j}$. The state of any node without incoming links must therefore be zero, and any node-state that receives only in-links from such zero-nodes also vanishes. Consider, on the other hand a single cycle in isolation. Each of its nodes inherits the states of their respective parent nodes, and therefore all cycle-nodes must have the same non-zero state. Now, if the cycle is embedded in a network, it has outgoing and incoming links. The incoming links cannot originate from the cycle, or else there would be nodes that are part of more than one cycle. Hence, they must come from vanishing nodes and cannot contribute to the cycle states. Therefore, also the nodes in the non-isolated cycle still have the same states. Finally, consider a node that has multiple in-links. Its state is simply the sum of the node-states pointing to it. Consequently, if a node has two, three, or four paths leading to it from the cycle (see node A or B in Fig. 1 right), its state is two times, three times, or four times the state of a cycle-node.

The magnitude of state *x*_*i*_ also captures the eigenvector centrality of the corresponding node *i* in the network. The eigenvector centrality is either defined iteratively or directly via Eq. (3), with *λ*_1_ equal to the largest eigenvalue^44^. The centrality *x*_*i*_ does not just measure the number of neighbors that *i* receives links from, but also accounts for their importance. So, a node with a high eigenvector centrality is one that receives links from very central nodes. The definition depends on the convention of the arrows for a given adjacency matrix. Equation (1) therefore gives rise to the eigenvector centrality in a dynamical way. Likewise, one may ask to which extent the quantization of the state vector changes for other centrality measures.

In practice, it is more useful to consider a reverse direction of the theorem, i.e., to predict one cycle from the eigenvector quantization. Indeed, the reverse direction is not true in general. When *λ*_1_ = 2, 3, …, it may happen that we observe the quantization too. However, for sparse networks, where *λ*_1_ is typically close to one, the reverse is true. In particular, this is the case for the Jain–Krishna model, which is presented in the following. In this case the eigenvector quantization can be used to determine the structural stability of and can provide a warning signal for impending collapse. Further, one may determine the path-distance of a given node to the last remaining cycle. In the following, we discuss several examples in which the quantization can be detected. In the first example, we focus on the aforementioned Jain–Krishna model where we demonstrate explicitly how well it works to actually predict a collapse.

### Co-evolutionary complex systems — Jain–Krishna model

We consider a simple model for the evolutionary dynamics of a toy ecosystem^33^. It was used to explain the rapid changes (punctuated equilibria) in species diversity and the network mechanisms behind it. In particular, the model shows large-scale collapses in diversity. We briefly describe the model, explain how our main result applies, and show how predictions about the average time to collapse can be obtained.

The Jain–Krishna model is a co-evolving network model that distinguishes between a fast time scale, at which populations evolve on a fixed interaction network *M*, and a slow time scale, at which the network evolves through selection and mutation of its species. The population *X*_*i*_ of species *i* is a proxy for its abundance. It evolves according to the linear catalytic dynamics given by (1). The relative abundance *x*_*i*_(*t*) converges to a fixed point *x*_*i*_. Thus, *x*_*i*_ is endogenously defined by the interaction network *M* and can be used as a measure for the fitness of species. The network is updated on the slow timescale as follows: the species with the smallest value of *x*_*i*_ (along with all its links) is eliminated. A new species is introduced, again labelled by *i*, which forms relations (links) with, on average, *m* randomly selected already existing species *j*. This is realised by independently assigning in-links to (*M*_*ij*_ = 1), and out-links from (*M*_*ji*_ = 1) the new species, *i*, both with the same probability *m*/(*N* − 1). In^33^, a range of *m* from 0.05 to 2 is considered. For *m* < 1, the model shows collapses. The Jain–Krishna model depends on two characteristic parameters: the average connectivity *m*, and the maximal diversity *N*. The observables in the model are the abundances *x*_*i*_ and the diversity given by the number of species that have a positive abundance,

$$S=\mathop{\sum }\limits_{i=1}^{N}\,\Theta ({x}_{i}),$$

where Θ is the Heaviside step function that equals 1 whenever its argument is greater than 0.

We briefly describe the mechanisms that lead to species diversification and to collapse. If there are no cycles in the network, most species must die out. We show this formally in the SI (case *λ* = 0 in Lemma 1), but here we provide an illustrative example. Consider only two species connected by a directed link. The receiving species grows proportional to the giving species. Asymptotically the former species gains a positive relative abundance, whereas the latter vanishes in relative terms. This argument can be extended to all cycle-free graphs, for which it can be shown that the only the species at the end of chains attain a positive relative abundance and the rest have asymptotically vanishing relative abundance (Lemma 1 of the *SI*). If there is at least one cycle, many species have positive abundance, they exist. Existing species are either part of a cycle, or have incoming paths from a cycle. In the network updating process the system may change, if a hitherto unfit species dies out and upon its re-introduction attaches itself to a fit (very abundant) species, thus becoming fit itself. The network updating process can also lead to the creation of new cycles, which can lead to a strengthening of the existing system^33^. Eventually, cycles can also break, whenever cycle-nodes become the least abundant species. This inevitably happens, since those species which are not part of a cycle either are already well adapted by influx from highly abundant species, or keep being replaced until they become well adapted. When all cycles break, all self-sustaining structures disappear, and the ecosystem collapses.

**Figure 2:**
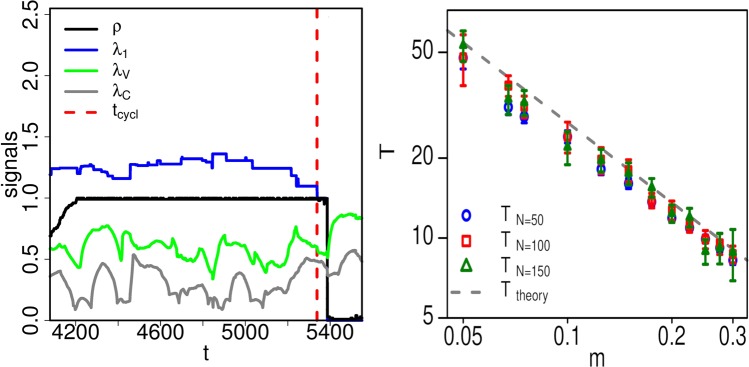
Left: sample run of the Jain-Krishna model. *ρ* is the fraction of populated nodes, *λ*_1_ is the largest eigenvalue. The time point *t*_*cycl*_ denotes the moment of entering the one-cycle phase (where the eigenvector quantization appears). We see that the collapse is preceded by the one-cycle phase. For comparison, we add two other precursor measures — volatility spectral radius *λ*_*V*_ and cross-correlation spectral radius *λ*_*C*_ (their definitions are in SI). We observe no signal *before* the collapse, the signal appears *after* the collapse. Right: comparison between numerical simulations (symbols) and the theoretical prediction of (4) (dashed line) of the expected time-to-collapse, 〈*T*〉, for various system sizes *N* ∈ {25, 100, 200}, and connectivities, *m* between 0.05 and 0.375. Simulation results follow the theoretical prediction closely, independent of system size and *m*.

Every collapse is always preceded by a phase where only one remaining cycle exists in the network, see (*SI*). This is demonstrated in Fig. 2 left, where a typical example of the Jain-Krishna collapse mechanism is shown. The detection of this last cycle thus becomes a warning signal for the impending collapse. Since the population dynamics is governed by (1), the quantization theorem applies for the Jain–Krishna model. Whenever the species vector *x* quantizes a warning signal is produced; the network now has only one cycle left and is potentially very close to collapse.

We can estimate the expected time-to-collapse once quantization is observed. This calculation is shown in detail in the (*SI*). Here we sketch the main idea: First, the probability of removing a cycle-node in the critical regime is *p* = *p*_*w*_*p*_*c*_, where *p*_*w*_ is the probability that the cycle-node belongs to the set of the weakest nodes, and *p*_*c*_ is the probability of choosing it from this set. The former can be approximated for sufficiently large sparse graphs (*m* < 1 and *N* ≫ 1) by the average network connectivity *m*, *p*_*w*_ ~ *m*. This is true because a new species only survives in the ecosystem if it receives at least one in-link, which happens with probability 1 − (1 − *m*/(*N* − 1))^*N*−1^ ~ *m*. Otherwise, it remains unpopulated and becomes the weakest species with vanishing abundance and will be replaced again. The latter, *p*_*c*_ can be expressed as the probability that a cycle of length *L*_*c*_ is formed among the *L* weakest nodes. This probability assumes its maximum and hence most likely value *p*_*c*_ ~ 1/*e* at *L*_*c*_ ~ *L*/*e*, as shown in the (*SI*) (*e* is Euler’s constant).

In summary, we obtain that the probability of removing a cycle-node is *p* = *m*/*e*. It is now possible to estimate the average life-time of the ecosystem once it enters the critical phase. The probability that the system collapses within the next *T* steps after the appearance of the quantization-based warning signal is given by *P*(*T*) = (1 − *p*)^*T*−1^*p*. The expected time-to-collapse in the critical phase can then be expressed as

4 $$\langle T\rangle =\mathop{\sum }\limits_{T=1}^{\infty }\,T\,P(T)=\frac{1}{p}\equiv \frac{e}{m}.$$

We explain the intuition behind this result. Since *m* is the rate at which a populated species is chosen for extinction, the factor 1/*m* gives the average time that it takes to remove a populated node. Further, since there is a chance 1/*e* that this node is a cycle-node, one needs to repeat the network update on average *e* times to pick out one cycle-node. Figure 2 right shows the comparison of the average times-to-collapse 〈*T*〉 for numerical simulations of the model and the theoretical predictions from (4). We run the model on R for a range of connectivities *m* and system sizes *N*, always for 10^7^ time steps. At each collapse, we record the time between the first observation of the quantization and the collapse. We average these times for all collapses in the run. The simulation results follow the theoretical predictions very well, irrespective of system size and *m*.

Finally, we compare the eigenvector quantization with other types of precursor signals. Well known precursor signals based on critical slowing down near the collapse point are given by the spectral radius of the lagged cross-correlation matrix *λ*_*c*_ and the spectral radius of volatility *λ*_*V*_^40^. For their definitions, see SI. We show the various precursor signals in Fig. 2 left, the cross-correlation spectral radius (green), and volatility spectral radius (grey). We observe no characteristic signals for the spectral radius before the collapse. Signals appear only after the collapse. We have run several independent simulation runs and never observed any signal for any of these two measures.

### Epidemic spreading in the SIS model

Let us now discuss a model where traces of the eigenvector quantization can be observed. We consider the classical *SIS* model on a sparse, directed (static) network *M*. It describes the spreading of a disease without immunity. Nodes can be either infected (*I*) or susceptible (*S*). An infected node can recover at a rate, *r*, or transmit the infection along a directed link to a susceptible neighbour at a rate, *β*. According to the individual-based mean-field approximation, the time evolution of the probability, *p*_*i*_, that a node *i* is infected is given by^23^

5 $$\frac{d}{dt}{p}_{i}=\beta \sum _{j}\,{M}_{ij}{p}_{j}(1-{p}_{i})-r{p}_{i}.$$

When the average disease prevalence, *p*_*i*_, is small, i.e., 1 − *p*_*i*_ ≈ 1, the dynamics of (5) becomes effectively linear and can be cast into the form of (1) by rescaling time *t*′ = *βt*, and setting Φ = *r*/*β*. Therefore, in this regime the theorem applies. However, because of these two approximations,  the linearity condition is not exactly met. We expect a deviation of *p* from the exact eigenvector quantization, which appears in the limiting vector *x* of the linear dynamics (1), whenever the network contains just one single cycle.

We run numerical simulations of the *SIS* model to compare the situation of single-cycle with the multi-cycle networks. The probability that a given node is infected, *p*_*i*_, is approximated by the average time it spends in the infected state, as a fraction of the total simulation time. We draw 10^3^ single- and multi-cycle graphs from the ensemble of Erdős–Rényi graphs of size *N* = 200 at the critical connectivity parameter, where the giant component would appear almost surely in the *N* → ∞ limit. We set *β* = 1, which corresponds to fixing a time-scale. Since for any finite value of *r* the infection dies out at some point (In the language of probability theory, this event happens almost surely, i.e., with probability one), we have re-initialized the model randomly, whenever this happened. For each network the duration of the simulation, including re-initializations, was 10^4^ × (*r* + *β*), which ensures the same average number of events per simulation for a fixed network size. The average infection time, *p*_*i*_, is then recorded for every node and rescaled by a factor *p*_min_, that shifts the location of the first maximum to 1.

**Figure 3:**
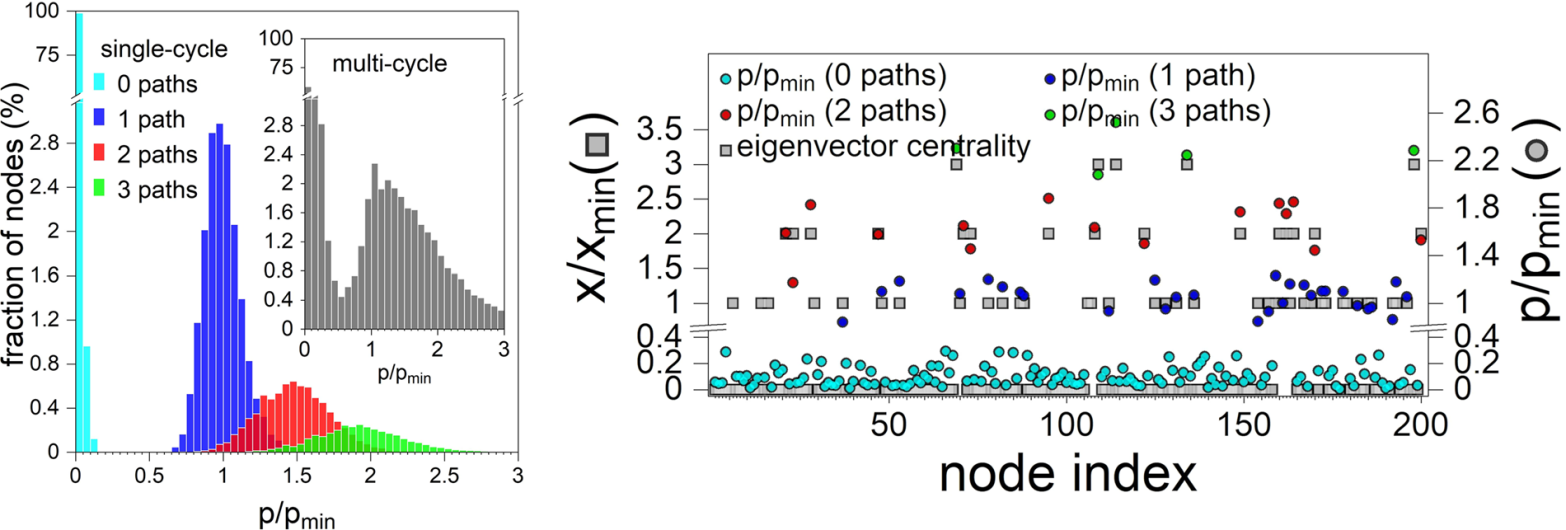
Comparison between the rescaled state vector, *x*/*x*_min_, and the rescaled average time of node infection, *p*/*p*_min_, which is taken as a proxy for the infection probability of a given node. Left: situation for a single-cycle network with *N* = 200 species that was randomly sampled from the Erdős–Rényi ensemble. Circles represent the average time of infection of the respective nodes, squares are the components of the rescaled eigenvector. The strong correlation between the two is apparent. Right: histogram of the average infection times for 10^3^ different networks of size *N* = 200 with single (main) and multiple cycles (inset). Note the presence of four peaks that correspond to nodes with zero, one, two, and three paths from the cycle. They are colored with cyan, blue, red, and green, respectively. No clustering around integer values is present for multi-cycle networks (inset). All networks are drawn from the Erdős–Rényi ensemble with at least one node being linked to the cycle via three paths.

In Fig. 3 left we show the comparison between the rescaled values *p*/*p*_min_, obtained from numerical simulations, and the rescaled eigenvector *x*/*x*_min_ for one randomly sampled Erdős–Rényi graph with a single cycle. The level structure of the infection duration is clearly visible and strongly correlated with the exact level structure of the eigenvector. In Fig. 3 right we show the histogram of *p*/*p*_min_ generated by sampling 10^3^ networks from the single-cycle ensemble and we compare it to the histogram of the multi-cycle ensemble in the inset. We choose a recovery rate *r* = 0.5. For *r* larger than *β* = 1, the infection has no chance to propagate through the cycle and the effect becomes less pronounced when compared to the *r* < *β* case, yet it is still visible. We color-coded the contributions according to the number of paths that lead to the contributing node from the cycle. One can clearly see the signature of the eigenvector quantization with four peaks in the single-cycle histogram, which indicates the average time of infection for nodes with zero, one, two, or three paths from the cycle. Quantization is not exact, as expected. Higher levels are not at integer values, but are shifted toward lower values. The reason for this is that the linearity assumption (5) breaks down. A node with two or three incoming paths from the cycle has a higher average duration of infection, because it can receive the infection in multiple ways. It cannot, however, receive the infection when it is already infected, which is reflected in the non-linear term *p*_*j*_(1 − *p*_*i*_) in (5) that forces the value of the infection probability *p*_*i*_ to be less than the multiplicity of paths.

### Generalizations of eigenvector quantization

Here we demonstrate that the eigenvector quantization is still approximately observable for mild generalizations of eigenvector centrality of binary networks. We focus on three examples — first is the Katz centrality, second the eigenvector centrality of (moderately) weighted networks and third a logarithmic variant of the eigenvector quantization in weighted networks.

#### Katz centrality

**Figure 4:**
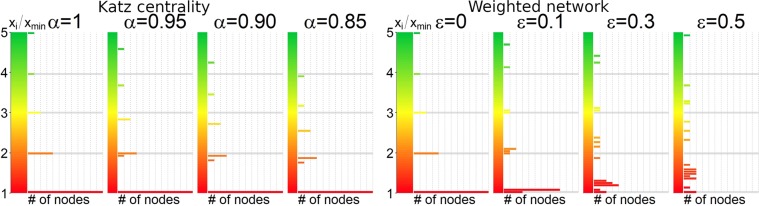
Left: histograms of the Katz centrality for single-cycle networks. The histograms show the number of nodes with a given value of ${x}^{(K)}/{x}_{{\rm{\min }}}^{(K)}$ for different values of *α* ∈ {1, 0.95, 0.9, 0.85} and *β* = 1. For *α* → 1, the same quantization can be seen. For *α* ≠ 1, while the level structure is still observed, these levels are no longer at integer values and some of them can be split up into sub-levels. Right: histograms of weighted networks. The single-cycle network from Fig. 1 right has been subjected to a weighting of its links. The weights are drawn from the interval [1 − *ε*, 1] uniformly at random. The histograms show the number of nodes with a given value of *x*/*x*_min_ for various values of *ε* ∈ {0, 0.1, 0.3, 0.5}. For *ε* = 0 the pure quantization can be seen. For *ε* > 0 the signature deteriorates.

Apart from the eigenvector centrality (3), one often considers the Katz centrality which is defined as

6 $${x}_{i}^{(K)}=\alpha \sum _{j}\,{M}_{ij}{x}_{j}^{(K)}+\beta ,$$

where 0 < *α* < (*λ*_1_)^−1^ and *β* > 0. The main difference between the Katz and the eigenvector centralities is the second constant term which gives any vertex (even those without any in-link) a score *β* in addition to the contribution from the centrality of its neighbours. We would like to show the possibility to observe an approximate eigenvector-quantization-like picture for the Katz centrality. In fact, the histograms of single-cycle networks Fig. 4(b) still show the level structure, but as long as *α* decreases, these levels are shifted from integer values and split up into sublevels.

#### Slightly weighted networks

We consider a moderately weighted network, by drawing the weights of the links from the interval [1 − *ε*, 1] uniformly at random, where *ε* is a noise parameter. When *ε* = 0 we recover the unweighted case. We investigate to which extend it is possible to distinguish the single-cycle networks from the multicycle networks. The noise parameter *ε* is varied from zero, the baseline model, to one, the fully random model.

The histograms of single-cycle networks still show the quantization peaks as one departs from the baseline scenario, but as *ε* increases, these peaks broaden and gradually lose their equidistance. For the single-cycle network in Fig. 4 left, we have looked at the histograms of *x* of weighted networks for various values of *ε*. The examples are seen in Fig. 4 right.

#### Example of the weighted network — Leslie model of population aging

The Leslie model is used in population ecology to understand age distributions by means of a linear reproduction dynamics. Even though it is mathematically trivial, it is relevant ecologically^45^. For reproductions in ecosystems, it is important that cycles are present. The life-cycles of animals that undergo metamorphosis and significant transformations are examples of these reproductive cycles. Salmons, for instance, pass through various stages that involve the adaptation from sweet water to saltwater environments, and large migrations^46^. If at any of these stages there is a bottleneck, e.g. closed migration routes, extensive fishery, or a major ecological catastrophe, then the reproduction cycle is broken and the entire population is at risk of extinction.

**Figure 5:**
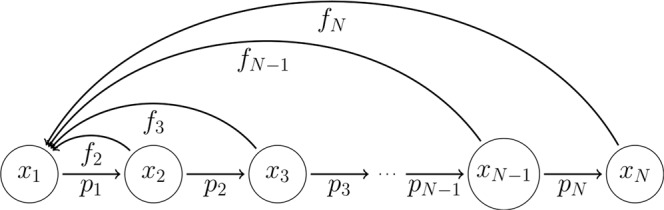
Illustration of the transitions in the Leslie model. There are *N* stages in the population (circles), each characterized by a population size *x*_*i*_. A percentage *p*_*i*_ ≤ 1 of the population in the *i*^*th*^ stage survives to the next stage *p*_*i*+1_, while the number of new individuals born into stage 1 for a given individual at stage *i* is *f*_*i*_.

We now describe how the theorem extends to the case of weighted links by means of the Leslie model. The population is divided into *N* stages, with population sizes *x*_1_(*t*), …, *x*_*N*_(*t*) at time *t*. The population at time *t* + 1 is obtained from the population at *t* by (see also Fig. 5)

7 $$\begin{array}{rcl}{x}_{1}(t+1) & = & \sum _{j > 1}\,{f}_{j}{x}_{j}(t)\\ {x}_{j}(t+1) & = & {p}_{j-1}{x}_{j-1}(t)\,{\rm{for}}\,j > 1.\end{array}$$

The first equation represents reproduction, where *f*_*j*_ is the number of offspring per individual at stage *j*. The second set of equations determines the survival (or aging) process, where *p*_*j*_ is the fraction of individuals that survive to the next stage. In matrix form, *x*(*t* + 1) = *Lx*(*t*), the components are given by *L*_*ij*_ = *f*_*j*_*δ*_*i*,1_ + *p*_*j*−1_*δ*_*i*−1,*j*_, where *δ*_*ij*_ is the Kronecker delta. Here *L* is called the *Leslie matrix* and (7) is a discrete version of (1).

Since the Leslie matrix *L* is weighted, one cannot use the quantization criterion directly. However, often one is interested to know whether there are one or more cycles: certain species reproduce only in a certain stage of their life. This means that there is only one non-zero *f*_*i*_, and *f*_*j*_ = 0, for *j* ≠ *i*. Let us also consider the case where all survival percentages are similar, such that *p*_*i*_ ~ *p*, ∀*i*.

Without loss of generality, we choose the reproductive stage to be the last one, *i* = *N*. If this is not the case, we focus only on the part of the system, which creates the reproduction cycle; the remaining stages do not feed back into the cycle and thus don’t contribute to the reproduction. For long times, the population is determined by the eigenvector equation, *Lx* = *λ*_1_*x*. In this case, it is possible to calculate *λ*_1_ and the corresponding eigenvector, ${\lambda }_{1}=p{(f/p)}^{\frac{1}{N}}$, and

$$x=({(\frac{f}{p})}^{\frac{N-1}{N}},{(\frac{f}{p})}^{\frac{N-2}{N}},\ldots ,{(\frac{f}{p})}^{\frac{1}{N}},1).$$

The logarithm of the population vector then reads

8 $$\log \,x=\frac{1}{N}\,\log \,\frac{f}{p}\cdot (N-1,N-2,\ldots ,1,0),$$

where we see a *logarithmic quantization*. Log-quantization also holds for more general networks, where we have all links with weight *p*, and a single reproduction link with weight, *f*. Moreover, each node should have only one in-link. For the unweighted network this corresponds to a situation where all links have the same population, *x*_*i*_ = *x*_*c*_. This scenario disappears for two or more reproduction cycles.

## Discussion

In many networked dynamical systems, directed cycles are crucial for self-sustaining feedback, autarchy, autocatalysis^24^, and consequently, are responsible for the structural stability of these systems. The breakdown of the last cycle in such systems is often associated with a drastic change of dynamical behavior—often collapse. We found that for linear networked dynamical systems the presence of a single cycle in an evolving network can be detected by a quantization phenomenon in the dynamical variables of the node states; the detailed information about the entire network is not necessary. Our method can be used to probe whether the system is in a highly vulnerable and unstable state, prone to collapse in the near future. Since structural features often evolve much slower than node features, this method can be used as an early warning signal for the collapse of networked dynamical systems. The advantage of this method is that it probes relevant structural features (last cycle) without relying on any information about the interaction network. We demonstrated the predictive quality of the method in a transparent model of co-evolutionary dynamics.

We expect to see traces of these cycles in data from a diverse range of applications, ranging from autocatalytic sets of RNA molecules^29^, gene regulatory networks^47^, small-sized ecosystems, to life-cycles of migrating animals, and those undergoing metamorphosis. Applications might also extend to socio-economic situations. Recently, it has been argued that autocatalytic cycles appear in technology^48^, and production networks^49^. A detailed data analysis of these systems might further confirm the relevance of the presented results. Note that it might not be the vector of populations, abundances, or revenues that exhibits the quantization in the single-cycle phase; it could show in the vector of deviations from its mean since the dynamics of many systems is linear around a given equilibrium.

The presented approach has limitations. We discuss the three most significant ones. For weighted networks the exact result does no longer hold. However, extensions and generalizations can be developed. The first extension to networks with link weights has been discussed in the last example of the previous section, which treats the population-age dynamics in the Leslie model with uniform survival and reproduction fractions. There we showed that a variant of the eigenvector quantization holds, where the logarithm of the node states is quantized in the presence of a single-cycle network. The second extension to moderately weighted networks, with weights taken at random from a small interval around one,  has been discussed in the second example of the previous section. The general case of arbitrarily weighted networks requires a more thorough treatment in further research. The other crucial assumption behind our approach is the linearity of the dynamics (1). We have shown in the epidemic spreading example that systems still retain signatures of quantization, even though the dynamics is not strictly linear. In this example, nonlinearity broadens the quantization peaks and for all positive levels (*n* ≥ 1) it reduces the inter-peak distance while retaining equidistance. However, even in linear systems, eigenvector quantization may cause false alarms, since in exceptional cases there may be an apparent quantization effect for very specific network-topologies with multiple cycles.

The linearity assumption implies that the eigenvector centrality is a reasonable observable of the system. In linear systems, the asymptotic relative state vector coincides with the eigenvector centrality. It is worth investigating to what extent other centrality measures, that arise from different (non-linear) dynamics, such as in the epidemic example, show an equivalent behaviour to the eigenvector quantization. We took first steps in this direction by investigating the Katz centrality for parameters that are close to the limiting case of the eigenvector centrality. A more comprehensive analysis is necessary in future work.

Finally, note that no assumptions are necessary about the dynamics of the network evolution, its rewiring, the deletion or addition of links or nodes. This fact is due to an immediate corollary of the Perron-Frobenius theorem — it follows that for linear dynamics, as in (1) the absence of cycles corresponds to a collapsed state because the vast majority of nodes cannot sustain themselves; their respective states vanish. Of course, one can conceive scenarios in which the no-cycle regime does not correspond to collapse states, or in which also other configurations are associated with collapse. It can also be that in such cases the collapsed regime can be reached directly without passing through the single-cycle phase.

## Supplementary information

Supplementary information.
