# Supplementary material for: Predicting collapse of adaptive networked systems without knowing the network
Source: Sci Rep. 2020 Jan 27;10:1223. doi: 10.1038/s41598-020-57751-y (PMC6985233; doi:10.1038/s41598-020-57751-y)
Supplement: Supplementary file 1 — Supplementary information. [file 41598_2020_57751_MOESM1_ESM.pdf]

Supplementary information: Predicting collapse of adaptive networked systems without knowing the network

Leonhard Horstmeyer[1,2,+], Tuan Minh Pham[1,2,+], Jan Korbel[1,2,+], and Stefan Thurner[1,2,3,4,+,*]

[1]Section for the Science of Complex Systems, CeMSIIS, Medical University of Vienna, Spitalgasse 23, A-1090, Vienna, Austria
[2]Complexity Science Hub Vienna, Josefstädterstrasse 39, A-1080 Vienna, Austria
[3]Santa Fe Institute, 1399 Hyde Park Road, Santa Fe, NM 87501, USA
[4]IIASA, Schlossplatz 1, 2361 Laxenburg, Austria
[*]To whom correspondence should be addressed. E-mail: stefan.thurner@meduniwien.ac.at
[+]these authors contributed equally to this work

ABSTRACT

Proof of Eigenvector Quantization Theorem

Theorem 1 (Eigenvector Quantization)**.** *Let M be a binary matrix with entries $M_{ij} \in \{0,1\}$ and diagonal entries $M_{ii} = 0$ for all $i \in \{1,\ldots,N\}$. Let G be the directed network with directed adjacency matrix M. Let $X(t) = (X_1(t),\ldots,X_N(t))$ be an N-dimensional state vector, whose components $X_i(t)$ evolve according to Eq. (1) from the main text. For all initial conditions $X(0)$, except for a set of Lebesgue-measure zero, the normalised vector $x(t)$, defined component-wise as $x_i(t) = X_i(t)/\sum_j X_j(t)$, converges to a stable fixed point $x := \lim_{t\to\infty} x(t)$, for which the following holds:*

Eigenvector Quantization: *Suppose G contains only one single cycle. Then any component x_i can be expressed as*

$$x_i = n_i x_{\min} , \tag{1}$$

where $x_{\min}$ is the minimal non-zero component and n_i is a natural number. The value of $x_{\min}$ is taken by the cycle-nodes, and the integer $n_i \geq 0$ is the number of directed paths that lead from cycle-nodes to node i. If there are no paths from cycle nodes, then $x_i = 0$.

Proof. Here we state some preliminary facts and definitions that allow us to outline and state the proof. First we define for each pair of nodes i and j of the graph G the quantity $\delta(i,j)$, which measures the length of the longest directed path from i to j. Furthermore we recall the eigenvalue equation for M

$$Mv = \lambda v$$

and denote by λ_1 the eigenvalue with the largest real part.The Perron–Frobenius theory states that $\lambda_1 = \rho(M) \geq 0$, where $\rho(M) \in \mathbb{R}$ is the real-valued spectral radius of M, and furthermore that $\lambda_1 = 0$ if and only if G contains no cycles[1]. Regarding the outline of the proof, we first show a Lemma proving the convergence of Eq. (1) from the main text to the Perron-Frobenius eigenvector by considering the cases $\lambda_1 = 0$ and $\lambda_1 > 0$ separately. Then we prove the eigenvector quantization.

Lemma 1. *For all initial conditions $X(0)$, except for a set of Lebesgue-measure zero, the normalised vector $x(t)$, defined component-wise as $x_i(t) = X_i(t)/\sum_j X_j(t)$, converges to a stable fixed point $x := \lim_{t\to\infty} x(t)$ which satisfies $Mx = \lambda x$.*

Proof of Lemma: **Case $\lambda_1 = 0$.** We will show that for almost all initial conditions the relative state vector $x(t)$ converges to a stable fixed point, which is a non-negative eigenvector of M. Let's denote $J := \{j | M_{jk} = 0 \, \forall k\}$ as the set of nodes without

incoming links. Provided that $X_j(0) > 0$ holds for all $j \in J$, then we can show by induction that

$$X_k^{(n)}(t) = \frac{t^n}{n!} \sum_{j:\delta(j,k)=n} X_j^{(0)}(0) + \mathscr{O}\left(t^{n-1}\right) \quad , \tag{2}$$

where the superscript in $X_k^{(n)}$ indicates that the node k is at a δ-distance n from the set J, so that $\delta(J,k) = n$. First, (2) holds for $n = 0$, because $dX_j^{(0)}(t)/dt = 0$ for any $j \in J$. Now, suppose that (2) holds for some n. We will show that it also holds for $n+1$. Integrating

$$\frac{d}{dt} X_\ell^{(n+1)} = \sum_{j:\delta(j,\ell)=1} X_j^{(n)}(t) \quad ,$$

and plugging equation (2) into it yields the desired result. This completes the proof by induction. Let $n_{\max} = \max_{j,k} \delta(j,k)$ be the length the longest directed path in the network and denote $k_{\max}$ those nodes at the ends of those paths. Then following (2) the states of the nodes $k_{\max}$ are of the order $\mathscr{O}(t^{n_{\max}})$. Upon normalization, the relative state vector reads $x_k(t) \simeq \frac{X_k^{(n)}}{\mathscr{O}\left(t^{n_{\max}}\right)} \simeq \frac{\mathscr{O}\left(t^n\right)}{\mathscr{O}\left(t^{n_{\max}}\right)} = \mathscr{O}\left(t^{n-n_{\max}}\right)$, which vanishes as $t \to \infty$ for any node $k \neq k_{\max}$. It can also be seen that any x, whose components vanish everywhere except on $k_{\max}$, where they assume non-negative values, are eigenvectors of M with eigenvalue $\lambda_1 = 0$, because $M_{jk_{\max}} x_{k_{\max}} = 0$ by definition of $k_{\max}$ having no outgoing links.

Case $\lambda_1 > 0$.

We consider the eigenspace V of λ_1. By considering the Frobenius normal form of the matrix M and applying ~~By~~ the Perron–Frobenius theorem to all its irreducible factors we know that the components of the eigenvectors ~~of~~ to λ_1 are all non-negative reals. Hence the eigenspace is a subspace of $\mathbb{R}^N$ and we define $V := \{v \in \mathbb{R}^N \mid Mv = \lambda_1 v\}$ and its orthogonal complement $W = V^\perp$ with respect to the Euclidean inner product $\langle \cdot, \cdot \rangle$. We pick an orthonormal basis $\{v^\alpha\}$ of V and a basis $\{w^\beta\}$ of W, which together form an orthonormal basis of $\mathbb{R}^N = V \bigoplus W$. Let us now consider an arbitrary solution $X(t)$ of Eq. (1), decomposed in that basis

$$X(t) = \sum_\alpha a_\alpha(t) v^\alpha + \sum_\beta b_\beta(t) w^\beta \quad . \tag{3}$$

We plug this decomposition into (**??**) and take the inner product with respect to the basis vectors $v^\alpha \in V$ and $w^\beta \in W$. Then we use the eigenvalue equation for λ_1 and the orthogonality $\langle v^\alpha, w^\beta \rangle = 0$ to obtain dynamical equations for the components a_α and b_β

$$\begin{aligned} \frac{d}{dt} a_\alpha(t) =& \lambda_1 a_\alpha(t) + \sum_\beta b_\beta(t) \langle v^\alpha, M w^\beta \rangle \\ \frac{d}{dt} b_\beta(t) =& \sum_{\beta'} \langle w^\beta, M w^{\beta'} \rangle b_{\beta'}(t) \quad . \end{aligned} \tag{4}$$

By defining the matrices B with components $B_{\beta\beta'} = \langle w^\beta, M w^{\beta'} \rangle$ and C with components $C_{\alpha\beta} = \langle v^\alpha, M w^\beta \rangle$ we can write the formal solution of the ODE:

$$a(t) = e^{\lambda_1 t} a(0) + e^{\lambda_1 t} \int_0^t ds\, e^{-\lambda_1 s} C e^{Bs} b(0) \tag{5}$$

$$b(t) = e^{Bt} b(0) \quad , \tag{6}$$

where $a(t)$ and $b(t)$ are the vectors with components $a_\alpha(t)$ and $b_\beta(t)$ respectively. First, we note from (5) and (6) that in any norm $\|a(t)\| \geq e^{\lambda_1 t} \|a(0)\|$, by the triangle inequality. Let us for the rest of the argument consider only vectors $X(0)$ with $\|a(0)\| > 0$. Then it holds in any norm that $\|b(t)\|/\|a(t)\| \leq \|e^{(B-\lambda_1)t} b(0)\|/\|a(0)\| \to 0$ as $t \to \infty$. For the 2-norm $\|\cdot\|_2$ it also holds that $\|X(t)\|_2^2 = \langle X(t), X(t) \rangle = \|a(t)\|_2^2 + \|b(t)\|_2^2$ and therefore

$$\frac{\|b(t)\|_2^2}{\|X(t)\|_2^2} = \frac{\|b(t)\|_2^2}{\|a(t)\|_2^2} \left(1 + \frac{\|b(t)\|_2^2}{\|a(t)\|_2^2}\right)^{-1} \xrightarrow[t\to\infty]{} 0 \,. \tag{7}$$

Then we consider $x_i(t) = X_i(t)/\|X(t)\|_1$ from (3). We note that the 2-norm and the 1-norm are equivalent, which means that there exist constants η and $\xi \geq \eta$, such that $\eta\|u\|_2 \leq \|u\|_1 \leq \xi\|u\|_2$ for any u in a finite dimensional space $\mathbb{R}^N$. Now, one may see from

$$\langle w^\beta, x_i(t)\rangle^2 = \frac{b^2_\beta(t)}{\|X(t)\|_1^2} \leq \frac{\|b(t)\|_2^2}{\|X(t)\|_1^2} \leq \frac{1}{\eta^2}\frac{\|b(t)\|_2^2}{\|X(t)\|_2^2} \xrightarrow[t\to\infty]{} 0\,,$$

that those components of the limiting vector x_i that are orthogonal to the eigenspace V vanish, precisely because $\langle w^\beta, x_i\rangle = \lim_{t\to\infty}\langle w^\beta, x_i(t)\rangle = 0$ for all $w^\beta \in W$.

We conclude that within the set of initial conditions $\Delta^{N-1} = \{x \in \mathbb{R}^N : \|x\|_1 = 1 \;\&\; x_i \geq 0\,\forall i\}$ there is a set of initial vectors $\mathscr{S}_0 = \{x_0 \in \Delta^{N-1} : \langle V, x_0\rangle \neq 0\}$ whose limiting vectors x have been shown to possess non-vanishing components only in the direction of V and no components in the direction of W, that is they belongs to the set $\Omega = \{x \in \Delta^{N-1} : \langle W, x_0\rangle = 0\}$. First of all $\mathscr{S}_0$ has full Lebesgue measure within Δ^{N-1}. Secondly, Ω is closed in $\mathscr{S}_0$, because V is closed in R^N (if it is a proper subspace, otherwise the lemma is trivial) and therefore $\Omega = V \cap \Delta^{N-1}$ is closed in $\mathscr{S}_0 = W^c \cap \Delta^{N-1}$, where W^c is the set-complement of W in $\mathbb{R}^N$. So points within ~~an~~some ε environment of the limiting set Ω converge to Ω, making it a stable limiting set and more precisely a set of stable limiting points. Lastly, all those limiting vectors x in V satisfy by definition the eigenvalue equation $Mx = \lambda x$ □

Eigenvector quantization: Let us now prove the main theorem. Let x be the unique eigenvector of M (Perron–Frobenius eigenvector) corresponding to $\lambda_1 = 1$ when there is only one cycle $\mathscr{C}$ in G. As $\mathscr{C}$ is the unique strongly connected component of G, only those nodes k, which are either in $\mathscr{C}$ or in the out-component $\mathscr{C}_{\text{out}}$ of $\mathscr{C}$, have $x_k > 0$[2]. Consider an arbitrary node $c \in \mathscr{C}$. Since there is no contribution to x_c from any of its upstream neighbours s that are in the in-component of $\mathscr{C}$ (as $x_s = 0$) and there is only one in-link from another $c' \in \mathscr{C}$ to c, $x_c = M_{cc'}x_{c'} = x_{c'}$.
Now let δ_i denote the maximal length of simple directed paths $\mathscr{P}_c$ from a cycle node c to a node $i \in \mathscr{C}_{\text{out}}$. One can show that

$$x_i = \sum_{\mathscr{P}_c} \left(M^{\delta_i}\right)_{ic} x_c = n_i x_c \quad . \tag{8}$$

where $n_i = \sum_{\mathscr{P}_c}\left(M^{\delta_i}\right)_{ic}$, by induction on the length levels, denoted by δ.
Step 0: For $\delta = 0$. Consider those nodes i with $\delta_i = \delta = 0$. These are precisely the cycle-nodes, which are at a distance 0 from the cycle. We have already shown $x_c = M^0 x_{c'}$ and $n_c = 1$ above.
Induction Step: Suppose (8) holds for all nodes i that have $\delta_i \leq \delta$ for some $\delta > 0$. For all nodes j with $\delta_j = \delta + 1$, we have

$$x_j = \sum_i M_{ji} x_i = \sum_{i:\delta_i=\delta} M_{ji} \sum_{\mathscr{P}_c}\left(M^{\delta_i}\right)_{ic} x_c = n_j x_c\,,$$

where $n_j = \sum_{\mathscr{P}'_c}\left(M^{\delta_i+1}\right)_{jc}$ and $\mathscr{P}'_c$ denotes such directed paths from c to j that are the concatenation of $\mathscr{P}_c$ with the directed edge from i to j. The relation (8) thus is proved.

Finally, as $\left(M^r\right)_{ic}$ yields the number of directed path of length r from any c to i[1]. Thus, by its definition, n_i equals to the number of directed paths that lead from those cycle-nodes to the node i. This together with the fact that $x_{min} = x_c$, as $n_c = 1$, $\forall c \in \mathscr{C}$ completes the proof of the eigenvector quantization. □

Now we consider some extensions to the theorem.

Jain–Krishna model

Proof that the collapse is preceded by a single cycle regime

Let us define a *collapse-keystone* as a node that belongs to all the cycle(s) in G. Then we have the following result:

Proposition 1. *Let v be a collapse-keystone node of a finite irreducible graph G with the adjacency matrix A, then either the largest eigenvalue λ_1 of G is equal to 1 or v is not the least populated node.*

This result is easily extended to general graphs by considering their decomposition into irreducible components via the Frobenius normal form. Let v be a collapse-keystone of a general graph G. The largest eigenvalue λ_1 of G equals the largest eigenvalue of all of its irreducible components. Since v must be part of all cycles, it is certainly inside this irreducible component and we conclude by Proposition 1 that either $\lambda_1 = 1$ for that component, and thus for the entire graph, or v is not the least populated node of that component and thus not of the entire graph either.

Proof. We prove this statement by showing its contraposition holds: *Suppose $\lambda_1 > 1$ and v is the least populated node, then there exists at least one cycle in G that does not contain v, i.e., v is not a collapse-keystone.*

Let $\mathscr{D}(v) := \{w \in \mathscr{V} : A_{wv} = 1\}$ be the set of downstream neighbours of v. Since v is the weakest, $x_w > x_v$ for all $w \in \mathscr{D}(v)$. Furthermore, from $\lambda_1 x_w = \sum_{s \neq v} A_{ws} x_s + x_v > \lambda_1 x_v$, we have $\sum_{s \neq v} A_{ws} x_s > (\lambda_1 - 1) x_v > 0$. This means that each node $w \in \mathscr{D}(v)$ has at least one in-link that does not come directly from v, that is $\forall w \in \mathscr{D}(v), \exists s \neq v : A_{ws} = 1$.

Since there is no cycle that does not contain v, any in-link A_{ws} to $w \in \mathscr{D}(v)$ must be downstream from v through another node $w' \in \mathscr{D}(v)$. Therefore, consider the subgraph $G(\mathscr{D}(v))$ of G which is constructed as follow: in $G(\mathscr{D}(v))$ a directed link $w_2 \to w_1$ is put if there exists a path from w_2 to w_1. By its construction, $G(\mathscr{D}(v))$ is an irreducible graph with at least $|\mathscr{D}(v)|$ directed links over $|\mathscr{D}(v)|$ nodes, hence $G(\mathscr{D}(v))$ must contain at least one cycle. This cycle corresponds to a closed directed path that does not contain v, so v is not a collapse-keystone. □

Other precursors

Here we mention some other precursors that are typically used in time-series analysis:

Spectral radius of the correlation matrix. Let us consider a multivariate correlation coefficient matrix with some lag k:

$$CC_{ij}(k) = \sum_t \frac{(x_i(t) - \mu_i)(x_j(t-k) - \mu_j)}{\sigma_i(t)\sigma_j(t-k)} . \tag{9}$$

The spectral radius λ_C of the matrix $CC(k)$ is considered as a precursor of a critical transition[3].

Spectral radius of volatility. We define the volatility matrix as

$\sigma^2_{ij}(k) = \sum_t (x_i(t) - \mu_i)(x_j(t-k) - \mu_j)$

Again, the spectral radius λ_V of σ^2 is considered as a precursor of a critical transition[3].

Expected Time-To-Collapse in the Jain–Krishna model

Proposition 2. *The average life time of the Jain–Krisna model in the critical phase is*

$$\langle T \rangle = \frac{e}{m} \quad , \tag{10}$$

with variance

$$\mathrm{Var}(T) = \frac{2e}{m}\left(\frac{e}{m} - 1\right) \quad . \tag{11}$$

Proof. Suppose that at present the system is in the single-cycle phase; then a crash may happen at any next time step. The probability $P^d(T)$ that the collapse happens at time T can be expressed as

$$P^d(T) = (1-p)^{T-1} p \quad , \tag{12}$$

where p is the probability that a cycle-node is removed from the single-cycle. This equation implies that until $T-1$ only those weakest nodes belonging to the periphery are removed, while one of the cycle-nodes is picked at T.

Let L denote the total number of the least fit species, those whose populations equal that of the cycle species. Since L consists of nodes having only one incoming link (otherwise they would not be the weakest), the chance by which a node of the graph belongs to the set L is

$$p_w = 1 - \left(1 - \frac{m}{N-1}\right)^{N-1} \quad . \tag{13}$$

For large sparse networks, we have $p_w \simeq m$. Further, among these L nodes, let L_c be the number of cycle-nodes, so $L_c \leq L$. If a node is randomly chosen from L, the chance that it is a cycle-node is

$$p_c = \frac{L_c}{L} \quad . \tag{14}$$

One can estimate this fraction using a combinatorial argument established by Gerbner et al.[4]: the number $n_L(k)$ of cycles of length k contained in a directed graph with L vertices $G(L)$ is given by, $n_L(k) \cong \left(\frac{L-1}{k-1}\right)^{k-1}$. The function $n_L(k)$ is strongly peaked at $\widehat{L_c} = \frac{L-1}{\mathrm{e}} + 1$, while it vanishes fast for any $k \neq \widehat{L_c}$. Hence among all possible cycles of length k that can be formed in $G(L)$, the most likely one has length $\widehat{L_c}$. Using $\widehat{L_c}$ to approximate L_c in (14), we obtain $p_c \simeq 1/e$. Finally, probability, p,

now can be defined as $p = p_c \cdot p_w = \frac{m}{e}$, since this probability equals the probability m, that one of those weakest nodes is chosen for removal, times the probability $1/e$, that it comes from the cycle. Note that the approximation, $p_w \simeq m$, becomes worse for small values of N. For this case, one should use the exact expression (13). Substituting $p = m/e$ in (12), we can calculate the expected time-to-collapse as $\langle T \rangle = \sum_{T=1}^{\infty} T\, P^d(T) = \sum_{T=1}^{\infty} T \left(1 - \frac{m}{e}\right)^{T-1} \frac{m}{e} = \frac{e}{m}$. An analog computation yields $\left\langle (T - \langle T \rangle)^2 \right\rangle = \frac{2e}{m} \left(\frac{e}{m} - 1\right)$. □

References

1. Cvetković, D., Doob, M. & Sachs, H. Spectra of graphs: theory and application. Pure and applied mathematics (Academic Press, 1980).

2. Newman, M. Networks: An Introduction (OUP Oxford, 2010).

3. Scheffer, M. et al. Early-warning signals for critical transitions. Nature **461 7260**, 53–9, DOI: 10.1038/nature08227 (2009).

4. Gerbner, D., Keszegh, B., Palmer, C. & Patkos, B. On the number of cycles in a graph with restricted cycle lengths. SIAM J. on Discret. Math. **32**, 266–279, DOI: 10.1137/16M109898X (2018). https://doi.org/10.1137/16M109898X.
